# *Chordodes mizoramensis* (Nematomorpha, Gordiida), a new species of horsehair worm from Mizoram, North-East India

**DOI:** 10.3897/zookeys.75.812

**Published:** 2011-01-12

**Authors:** Andreas Schmidt-Rhaesa, Lalramliana

**Affiliations:** 1 Zoological Museum, University Hamburg, Martin-Luther-King-Platz 3, 20146 Hamburg, Germany; 2 Department of Zoology, Pachhunga University College, Aizawl, Mizoram, India

**Keywords:** Nematomorpha, Gordiida, *Chordodes*, new species, hairworm, cuticle

## Abstract

Chordodes mizoramensis, a new species of freshwater gordiid horsehair worm, is described from Mizoram, NE India on the basis of scanning electron microscopic and morphometric studies. The new species can be distinguished from its congeners in that the apical filaments of the crowned areoles are branched several times, a pattern that has not been observed in other species. An additional distinguishing character is that it has more bulging areoles, which are distributed among simple areoles alone or in groups, do not form clear patterns.

## Introduction

About 350 species of freshwater horsehair worms (Nematomorpha: Gordiida) are currently known. Of these, only 14 species (plus an additional undetermined Gordius sp.) have been reported from India (Schmidt-Rhaesa and Yadav 2004). Considering the size of India and the diversity of habitats, this number appears to be only a fragment of the existing gordiid diversity. We describe here a new species of the genus Chordodes Camerano, 1897.

Chordodes includes mainly tropical and subtropical species. All horsehair worms are parasites of arthropods, which leave their host for reproduction (Hanelt et al. 2005). Praying mantids form the main group of final hosts for species of Chordodes (see Schmidt-Rhaesa and Ehrmann 2001). Recently, the terminology for cuticular structures was unified and an overview and key were developed (Schmidt-Rhaesa et al. 2008). Characteristic for Chordodes species is, in comparison with other freshwater genera, the diversity of cuticular structures. The cuticle is often structured into polygonal or roundish structures named areoles. While in other genera not more than two different types of areoles can be recognized, species of Chordodes may exhibit up to seven types.

## Material and methods

The specimens investigated were preserved in 70% ethanol, directly after their emergence from the host, an undetermined praying mantis. Pieces about 1 mm long were cut from the mid-body region of each worm. These and the entire posterior ends were prepared for Scanning Electron Microscopy (SEM). The pieces were dehydrated in an increasing ethanol series, critically point dried and coated with gold in a sputter coater. Observation took place using a LEO SEM 1524 under 10 kV. Digital images were taken.

## Results

### Chordodes mizoramensis

sp. n.

urn:lsid:zoobank.org:pub:5A212538-A6FC-4C30-BB1F-B56169437AF6

Figs 1 [Fig F2] 3 

#### Type locality.

Mamit Village, Mamit District, Mizoram, India, 23°54'54.94"N, 92°29'16.75"E. Collected July 21, 2010 by Lalramliana and Remsangpuia.

#### Holotype.

Male specimen from the type locality emerged from Hierodula sp. (type-host). Deposited in the Zoological Museum in the Department of Zoology at Pachhunga University College, Aizawl-Mizoram, India, accession number PUCZM - A/V/1114.

#### Paratype.

Male specimen from the same host specimen and same locality as the holotype. Deposited in the Zoological Museum in the Department of Zoology at Pachhunga University College, Aizawl-Mizoram, India, accession number PUCZM - A/V/1115.

#### Host.

**Figure 1.**
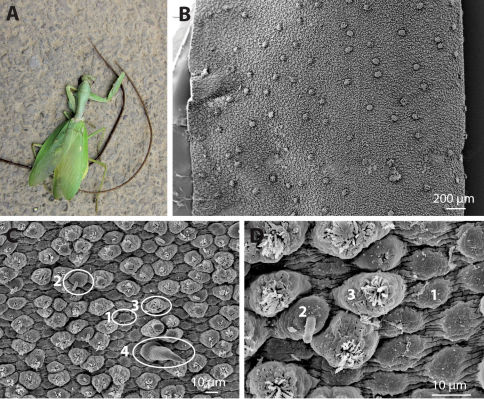
Chordodes mizoramensis, sp. n. **A** Hierodula sp., with both specimens of hairworm emerging from it. The darker specimen is the holotype **B** Overview of a stretched piece of cuticle from an entire section in the mid-body region, showing the distribution of areoles. Elevations are clusters of crowned and circumcluster areoles **C** Cuticle with simple (1), tubercle (2), bulging (3) and thorn (4) areoles **D** Magnification of the structure of simple (1), tubercle (2) and bulging (3) areoles. **B–D** from paratype, SEM.

Both specimens emerged from one specimen of Hierodula sp. (Mantodea) (Fig. 1A).

#### Etymology.

The name refers to the region in which the new species was found, Mizoram in NE India.

#### Description.

The holotype is 200 mm long, with a diameter of 1.3 mm in the mid-body region. Towards the posterior end, the diameter decreases to about 0.7 mm at the level of the cloacal opening. The anterior end is also tapered. The paratype is 265 mm long and has a diameter in the mid-body region of 1.5 mm;, at the level of the cloacal opening the diameter is 0.79 mm. The frontal tip in both specimens is white, whereas the remaining body is medium brown. A pattern of darker patches (the “leopard pattern”) is present in both specimens; in the holotype this is more pronounced than in the paratype.

The cuticle contains six types of areoles (areoles are elevated cuticular structures), for which the terminology of Schmidt-Rhaesa et al. (2008) will be applied. Most abundant are simple and bulging areoles (Fig. 1B–D). Simple areoles are quite flat semicircular elevations with a rough surface but no further structure (Fig. 1D). Bulging areoles are more elevated and carry a small tuft of very short bristles on top (Fig. 1C, D). Compared with other species, bulging areoles are quite abundant; they are distributed among simple areoles alone or in groups, without forming clear patterns (Fig. 1C). Tubercle areoles regularly occur among the simple and bulging areoles (Fig. 1C, D), as rarely do thorn areoles (Fig. 1C). Tubercle areoles carry a finger-like process on top; thorn areoles have a strong thorn on top of a broader basis.

**Figure 2. F2:**
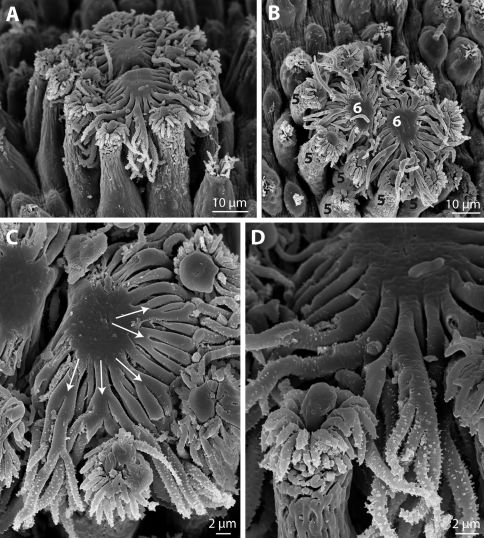
Chordodes mizoramensis, sp. n. **A–D** Crowned (6 in B) and circumcluster areoles (5 in B), **C** and **D** at magnifications demonstrating the branching of apical filaments. **A–D** from paratype, SEM.

Characteristic for species of Chordodes are crowned areoles, which carry a crown of apical filaments on an elevated “stem”. Crowned areoles occur in pairs and are surrounded by so-called circumcluster areoles (Fig. 2A, B). This last type resembles the bulging areoles, but is longer (as elevated as the crowned areoles) and more slender (Fig. 2A, B). It also carries an apical tuft of short bristles, some of which can be slightly branched. Several circumcluster areoles have a more or less central “plug” among the apical bristles (Fig. 2A–D). This “plug” is variable in shape, in some cases appearing as a drop-like structure emerging from the centre of the areole, but in others it is a broader, more voluminous structure. One pair of crowned areoles occurs in the centre, between the circumcluster areoles. Each crowned areole has a flat, smooth surface, with filaments emerging from the margin, except for the region where both areoles face each other (Fig. 2A–C). The filaments spread flat from the central surface and project between the circumcluster areoles. Their length is about 25 µm. Most filaments divide several times, forming multiple branches (Fig. 2A–D). Only one type of crowned areoles could be found.

**Figure 3.**
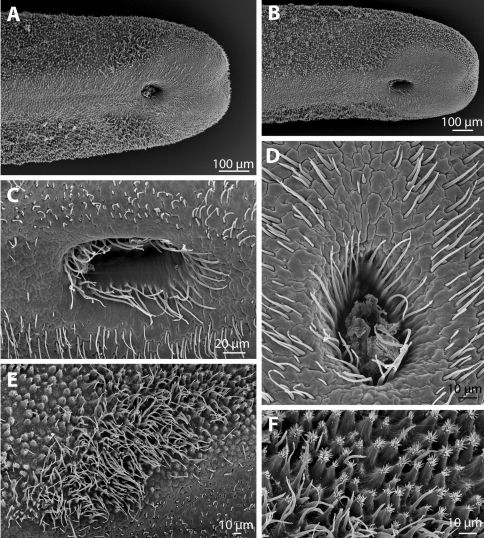
Chordodes mizoramensis, sp. n. **A–F** Posterior end. **A, B** Ventral view of posterior end of holotype (**A**) and paratype (**B**) showing the distribution of areoles and the ventral cloacal opening **C, D** Cloacal opening of the holotype (**D**) and paratype (**C**), showing circumcloacal bristles and further bristles in the region around the cloacal opening **E** Field of bristles anterolateral of the cloacal opening (holotype) **F** Form of areoles posterior to the field of bristles (paratype). **A–F** SEM.

The posterior end of the males is rounded, and a small median incision may be present (Fig. 3A, B). An approximately 150 µm broad ventral strip is free of areoles of the types described above, but forms polygonal or interdigitating compartments with a smooth surface (Fig. 3A, B). This smooth region extends around the ventral cloacal opening, which is about 200 µm anterior of the posterior margin of the worm. The cloacal opening is oval, with a number of long, fine bristles, the circumcloacal bristles, present in a ring emerging approximately 10 µm below its surface (Fig. 3C, D). In the region around the cloacal opening are further bristles; these are abundant and variable in length (Fig. 3C, D). The areoles described above are replaced at the posterior end, at least on the lateral sides, by elevated, conical areoles with an apical tuft of bristles (Fig. 3F). These areoles may represent bulging areoles, but are distinctly pointed apically and more abundant. In a region anterolateral to the cloacal opening is, in the region with areoles, an oval region with more bristles (Fig. 3A, B, E). These are very dense, appear to be all unbranched and have a lengths of up to about 30 µm.

## Taxonomic remarks

With about 90 described species, Chordodes is distributed in tropical and subtropical regions worldwide (Schmidt-Rhaesa et al. 2008). From India, four Chordodes species have been reported: Chordodes liguligerus Römer, 1895, Chordodes pollonerae Camerano, 1912, Chordodes siamensis Camerano, 1903 and *C*. cf. *furnessi* Montgomery, 1898 (see Montgomery 1898, Camerano 1903, 1908, 1912, Römer 1895, Schmidt-Rhaesa and Yadav 2004). Several more species of this genus can be expected to occur in India. Praying mantids are the main host group for species of Chordodes (see Schmidt-Rhaesa and Ehrmann 2001).

The types of areoles present on the cuticle of Chordodes mizoramensis sp. n. represent the “usual” set of areoles present in other Chordodes species, but there are some notable differences. Bulging areoles occur in some, but not all Chordodes species (see Schmidt-Rhaesa et al. 2008). They are distinctly more abundant in Chordodes mizoramensis than in other species. Crowned areoles surrounded by circumcluster areoles is also a common pattern, but there is no species in which a branching of the apical filaments has been described.

Several Chordodes species have two types of crowned areoles; those with distinctly longer apical filaments are present along the ventral and sometimes also the dorsal mid-line (see Schmidt-Rhaesa et al. 2008). In Chordodes mizoramensis, only one type of crowned areoles could be found. However, crowned areoles appear to be a sexually dimorphic character, with females showing both types of crowned areoles, whereas in males the differences appear to be much less distinct or absent (see, e.g. Schmidt-Rhaesa 2002 for Chordodes queenslandi Schmidt-Rhaesa, 2002 or De Villalobos et al. 2004 for Chordodes brasiliensis Janda, 1894). Therefore, it cannot be excluded that the females of Chordodes mizoramensis also exhibit such a dimorphism.

The male posterior end of the new species corresponds, as far as is known, in general with those of the males of other Chordodes species. However, the shape of the areoles on the posterior end (conical, with tuft of bristles on top) may be peculiar to Chordodes mizoramensis.

In summary, Chordodes mizoramensis exhibits some unique features, which justify its description as a new species.

In the key provided by Schmidt-Rhaesa et al. (2008), the new species must be placed in the following way: 1-4-5-7-9-18-19-27-29-31-32-33; under 33 there must be an extra line saying:

“crowned areole filaments branched  Chordodes mizoramensis”

## Supplementary Material

XML Treatment for Chordodes mizoramensis

## References

[B1] Camerano L (1903) Nuove specie di Gordii del Basso Siam. Boll. Mus. Zool. Anat. Comp. R. Univ. Torino 18:1-3

[B2] Camerano L (1908) Gordiens du Musée Indien. Rec. Indian Mus. 2: 112–117

[B3] Camerano L (1912) Gordiens du Musée Indien. Rec. Indian Mus. 7: 215–216

[B4] De Villalobos C Zanca F Schmidt-Rhaesa A (2004) New data on South American species of *Chordodes* (Nematomorpha). Arq. Mus. Nac. Rio de Janeiro 62:375-386

[B5] Römer F (1895) Die Gordiiden des Naturhistorischen Museums in Hamburg. Zool. Jb. Syst. 8: 790–803

[B6] Schmidt-Rhaesa A (2002) Australian species of *Chordodes* (Nematomorpha) with a description of two new species, remarks on the genus and its life history. J. Nat. Hist. 36: 1569–1588

[B7] Schmidt-Rhaesa A de Villalobos LC Zanca F (2008) Summary of *Chordodes* species (Nematomorpha, Gordiida), with a discussion of their diagnostic characters. Verh. Naturwiss. Ver. Hamburg 44:37-114

[B8] Schmidt-Rhaesa A Ehrmann R (2001) Horsehair worms (Nematomorpha) as parasites of praying mantids. Zool. Anz. 240: 167–179

[B9] Schmidt-Rhaesa A Yadav AK (2004) First report of *Chordodes* cf. *furnessi* (Nematomorpha) from a praying mantid in India with a note on Indian nematomorph species. Curr. Sci. 86: 1023–1027

